# Mechanistic insights into psoriasis and type 2 diabetes mellitus comorbidity – Implications for treatment: A review

**DOI:** 10.17305/bb.2026.13484

**Published:** 2026-01-21

**Authors:** Ling Ouyang, Zhanzong Li, Yunhong Zeng

**Affiliations:** 1 Community Health Management Department, Shenzhen Guangming District People’s Hospital, Shenzhen, Guangdong, China

**Keywords:** Psoriasis, type 2 diabetes mellitus, comorbidity, pathogenesis, treatment.

## Abstract

Psoriasis is a chronic systemic inflammatory disease primarily affecting the skin, yet it is increasingly recognized for its systemic implications, particularly its strong association with type 2 diabetes mellitus (T2DM). This review synthesizes recent mechanistic and clinical evidence to elucidate the shared pathways linking psoriasis and T2DM, as well as to explore therapeutic strategies for this comorbidity. We conducted a narrative review of studies published between January 2020 and October 2025, encompassing preclinical models, clinical trials, and high-quality reviews that address pathogenesis and treatment. Key findings indicate that shared genetic loci and molecular pathways, including nuclear factor kappa-light-chain-enhancer of activated B cells (NF-κB) signaling, the IL-23/Th17 axis, and mitochondrial dysfunction associated with the activation of the cyclic GMP-AMP synthase (cGAS)-stimulator of interferon genes (STING) pathway, contribute to both cutaneous inflammation and systemic metabolic dysregulation. Additionally, adipokine imbalances and chronic low-grade inflammation exacerbate insulin resistance and psoriatic skin pathology. Therapeutically, IL-17/IL-23 inhibitors, metformin, glucagon-like peptide 1 (GLP-1) receptor agonists, and other immunomodulatory strategies demonstrate potential in addressing both dermatologic and metabolic features. These insights reinforce the notion of psoriasis as a systemic disorder with significant metabolic consequences, highlighting the need for integrated, multidisciplinary management. Future research should concentrate on precise gene-environment interactions, biomarker validation, and the development of treatments that simultaneously target both skin and metabolic pathology to advance precision medicine for patients with psoriasis-T2DM comorbidity.

## Introduction

Psoriasis is a chronic systemic inflammatory disease primarily affecting the skin. It is characterized by inflamed, scaly plaques that cause considerable distress due to their visibility and associated symptoms, such as pruritus and pain [1]. The global prevalence of psoriasis ranges from 0.1% to 3%, with incidence rates being similar for both men and women [2]. Emerging evidence suggests that psoriasis is not merely a cutaneous disorder, but also has systemic implications [3].

One notable association is between psoriasis and type 2 diabetes mellitus (T2DM), which has received considerable attention. A retrospective cohort study indicated that hyperglycemia is an independent predictor of severe psoriasis recurrence [4]. In murine models, sustained glucose intake has been shown to exacerbate psoriasiform dermatitis, an effect that can be ameliorated by oral metformin [5]. Furthermore, psoriasis may elevate the risk of developing T2DM through systemic inflammatory mechanisms, reinforcing the notion of psoriasis as a systemic disease that disrupts metabolic homeostasis [6–8]. Several studies have identified overlapping distribution patterns and a positive genetic correlation between psoriasis and T2DM, bolstering the evidence for their link [9]. Additionally, the co-occurrence of psoriasis and diabetes has been associated with an increased risk of viral infections and retinal vein occlusion (RVO) [10].

Both psoriasis and T2DM are chronic conditions that significantly impair patient quality of life. Understanding the mechanisms driving both disorders and developing targeted management strategies are critical priorities. Emerging evidence points to a bidirectional relationship in which psoriasis, T2DM, and obesity exacerbate one another [11]. This interplay is rooted in shared pathological processes, including obesity, insulin resistance, and persistent systemic inflammation [12, 13]. In this review, we synthesize recent mechanistic findings from preclinical studies, clinical trials, and real-world datasets. We discuss the pathogenesis underlying the psoriasis-T2DM comorbidity and highlight therapeutic approaches targeting these shared disease mechanisms.

## Methods

We conducted a comprehensive search of PubMed, Google Scholar, and Web of Science for studies published between January 1, 2020, and October 1, 2025. The search terms included: psoriasis, psoriatic, type 2 diabetes mellitus, and diabetes mellitus. Inclusion criteria were as follows: (1) studies examining the relationship between psoriasis and T2DM; (2) clinical or experimental studies reporting complete data; (3) high-quality review articles providing comprehensive background information and references; (4) articles published in peer-reviewed journals. Exclusion criteria included: (1) studies that did not directly address the mechanisms or treatment of psoriasis and T2DM; (2) conference papers and studies with incomplete data; (3) studies of poor quality or inadequate experimental design. It is important to note that this review is narrative in nature, and no formal risk-of-bias assessment was conducted.

## Shared genetic susceptibility and mitochondrial dysfunction

Trans-disease meta-analysis (TDMA) has identified shared susceptibility loci for psoriasis and T2DM, including 2p14 (*P* ═ 9.6×10^--9^), 10q24.31 (*P* ═ 1.0×10^--9^), 11q13.1 (*P* ═ 1.0×10^--11^), and 17q21.2 (*P* ═ 1.5×10^--9^) [14]. Several proteins encoded by these loci, such as actin related protein 2 (ACTR2), endoplasmic reticulum (ER) lipid raft associated 1 (ERLIN1), and beclin 1 (BECN1), interact with tumor necrosis factor (TNF) receptor associated factor 6 (TRAF6) in the nuclear factor kappa-light-chain-enhancer of activated B cells (NF-κB) signaling pathway [14]. This interaction suggests that NF-κB may serve as a critical molecular link between psoriasis and T2DM. Additionally, bioinformatics analysis of data from the Gene Expression Omnibus (GEO) database has identified 62 shared hub genes between psoriasis and T2DM [15]. These genes include inflammatory mediators such as interleukin (IL)-1β and IL-17A, as well as several members of the S100A family. Metabolic regulators such as arginase 1 (ARG1) and aldo-keto reductase family 1 member B10 (AKR1B10) are also included. Notably, the late cornified envelope (LCE) gene cluster is well recognized for its association with psoriasis [16], demonstrating significant pharmacogenetic interactions. Dipeptidyl peptidase-4 inhibitors (DPP-4i), commonly used to manage T2DM, have been shown *in vitro* to enhance the expression of LCE-1C and LCE-3C in keratinocytes. In three-dimensional skin models, these drugs also increase LCE-2 protein levels [16]. This upregulation of LCE expression may partially compensate for psoriasis-related LCE-3B/C deletions, potentially aiding in the restoration of the epidermal barrier and providing a protective effect in psoriasis lesions.

In psoriasis patients treated with methotrexate, those with the rs12025144 single nucleotide polymorphism (SNP) GG genotype exhibit a higher incidence of diabetes and a diminished therapeutic response [17]. This observation suggests that this genotype may delineate a metabolic subtype of psoriasis characterized by underlying metabolic dysfunction. Furthermore, the ST6GAL1 gene encodes a sialyltransferase that modulates both immune and metabolic processes [18]. Bioinformatic analyses indicate that the rs6783836-T allele of ST6GAL1 is associated with lower hemoglobin A1c (HbA1c) levels, reduced lymphocyte counts, and a decreased risk of psoriasis [18].

Mitochondrial dysfunction represents another crucial link between psoriasis and T2DM. Whole mitochondrial genome sequencing conducted on 98 individuals revealed that the M haplogroup, characterized by a mitochondrial cytochrome b (MT-CYB) variant, is associated with a fourfold increase in psoriasis risk (odds ratio, OR = 4.0, *P* ═ 0.003). This association may be linked to reduced activity of the oxidative phosphorylation (OXPHOS) complex [19]. Conversely, haplogroups R0 and J are associated with a decreased risk of T2DM (OR = 0.28, *P* ═ 0.007). The T16189C variant within these haplogroups correlates with elevated fasting insulin levels and increased insulin resistance [19]. Functional studies in Arab cohorts have identified eight novel mitochondrial mutations [13]. These non-synonymous mutations impact key subunits of the respiratory complexes essential for energy production, specifically in Complexes I (ND2, ND4, ND5), III (CYB), and V (ATP6). Such mutations may hinder adenosine triphosphate (ATP) synthesis while increasing reactive oxygen species (ROS) production. Excessive ROS may damage insulin-sensitive β-cells, exacerbating both cutaneous inflammation and metabolic dysregulation [13].

Mendelian randomization (MR) studies conducted in European populations have demonstrated a positive genetic correlation between psoriasis and T2DM (genetic correlation, RG = 0.19, *P* ═ 3 × 10^--3^). However, there is insufficient evidence to establish a direct causal relationship between the two conditions [20, 21]. The observed association may be mediated by factors such as obesity and systemic inflammation, among others. Consequently, the relationship cannot be simplified into a singular causal pathway within MR analyses. These findings indicate that the connection between psoriasis and diabetes likely reflects intricate interactions among genetic predispositions, environmental influences, and clinical risk factors. Further mechanistic studies are necessary to elucidate these pathways.

## IL-17: A central immunometabolic mediator

Clinical research has shown that patients with psoriasis and concomitant T2DM exhibit significantly elevated levels of IL-17, IL-23, and tumor necrosis factor alpha (TNF-α) in affected skin [22]. Under stress conditions, skin dendritic cells and keratinocytes release IL-23, which subsequently activates T helper 17 (Th17) cells and γ δ T cells, leading to the secretion of IL-17A, IL-17F, and IL-22 [23]. In basic research, γ δ T cells in normal mouse skin did not express C-C chemokine receptor 6 (CCR6) [24]. However, in imiquimod (IMQ)-induced psoriasis mouse models, a functional subset of γ δ T cells emerged, characterized by the expression of both CCR6 and IL-17A. The ligand C-C motif chemokine ligand 20 (CCL20) interacted with T cell receptor (TCR) signaling, significantly enhancing IL-17A production, while exerting minimal influence on TNF-α levels [24]. Single-cell transcriptomic analyses revealed that epidermal CCR6^+^ γ δ T cells in psoriatic lesions expressed elevated levels of IL-17F, IL-22, and retinoic acid receptor-related orphan receptor alpha (RORα) [24].

**Figure 1.**
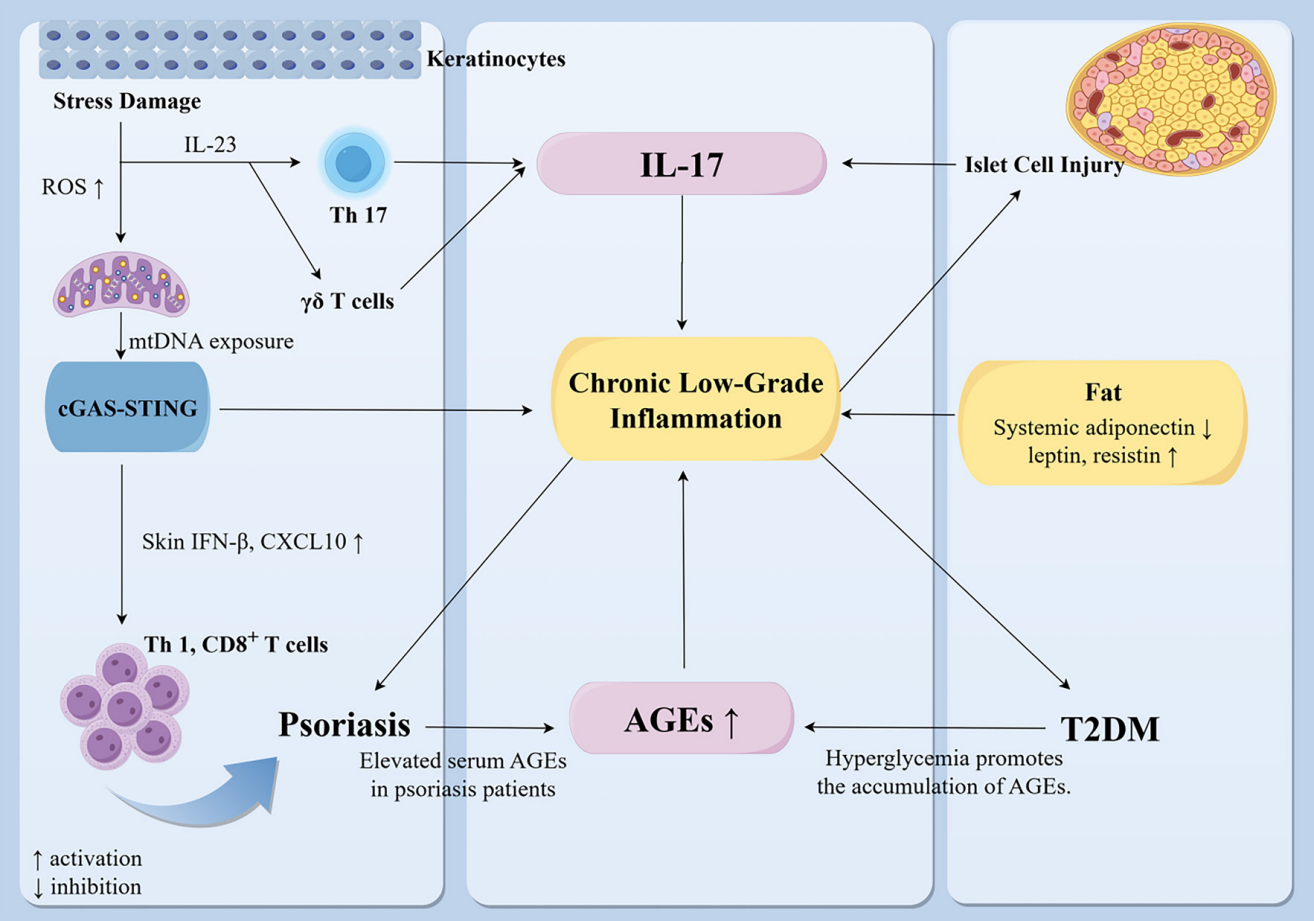
**Partial mechanisms underlying the co-occurrence of psoriasis and T2DM.** Under stress, skin dendritic cells and keratinocytes release IL-23, which activates Th17 cells and γ δ T cells. These cells subsequently secrete IL-17A, IL-17F, and IL-22 [23]. Additionally, damaged pancreatic β-cells also produce IL-17, as demonstrated in animal studies [27]. Together with other cytokines, this process contributes to a systemic low-grade inflammatory state. Co-stimulation of human keratinocytes with palmitic acid and imiquimod induces the release of mtDNA into the cytoplasm. The cytosolic DNA is recognized by cGAS, which activates STING and promotes its oligomerization. This cascade results in increased expression of IFN-β and chemokines, such as CXCL10, which recruit Th1 cells and cytotoxic CD8^+^ T cells to the epidermis, further amplifying local inflammation [33] (cell and animal studies). Elevated serum levels of AGEs are observed in psoriasis patients, with hyperglycemia in T2DM exacerbating circulating AGEs [34]. Furthermore, obesity exacerbates systemic low-grade inflammation [8]. Collectively, this chronic low-grade inflammation serves as a common pathological basis for both psoriasis and T2DM. Abbreviations: T2DM: Type 2 diabetes mellitus; Th17: T helper 17; γ δ T: Gamma delta T; β-cells: Beta cells; mtDNA: Mitochondrial DNA; cGAS: Cyclic GMP-AMP synthase; STING: Stimulator of interferon genes; IFN-β: Interferon-beta; CXCL10: C-X-C motif chemokine ligand 10; AGEs: Advanced glycation end-products. *This figure was created using Home for Researchers.*

Recent studies have underscored the significance of IL-17 in metabolic disorders, including type 1 diabetes mellitus (T1DM) and T2DM [25–27]. IL-17 is a pivotal proinflammatory cytokine and a primary effector of Th17 cells [28, 29]. Th17 cells are instrumental in driving autoimmune responses and play a crucial role in the pathogenesis of psoriasis. IL-23 facilitates the differentiation of Th17 cells and stimulates IL-17 release. Subsequently, IL-17 may incite systemic inflammation that adversely affects the function of remote organs, including pancreatic β-cells [30]. Notably, IL-17 expression is significantly diminished in insulin-deficient pancreatic islets, indicating a potential relationship between IL-17 levels and the functional integrity of β-cells [27]. Investigations utilizing formalin-fixed paraffin-embedded (FFPE) pancreatic tissue from 21 human cadaveric donors have revealed that pancreatic endocrine cells, encompassing both β- and α-cells, may actively produce IL-17 rather than merely serving as immune targets [27]. Immunofluorescence analysis has confirmed IL-17 expression within islets from patients with T1DM and T2DM, primarily localized in β- and α-cells rather than in CD45+ immune cells [27]. Collectively, these findings suggest that IL-17 production may persist despite an overall decline in its levels coinciding with progressive β-cell loss. Under metabolic or immune stress, islet cells can exacerbate local inflammation through IL-17 secretion. Elevated concentrations of circulating TNF-α and IL-17, in conjunction with locally heightened IL-23, may perpetuate cutaneous inflammation and contribute to systemic insulin resistance [31].

At the systemic level, patients with psoriasis-associated metabolic syndrome (PSO-MS) exhibit significantly higher serum IL-17A concentrations (2108 pg/mL) compared to those without metabolic abnormalities (162 pg/mL, *P* ═ 0.009) [32]. In contrast, IL-23 levels do not differ between these groups. This observation underscores the significance of IL-17, although the absolute values and fold changes may vary based on the assay platform and detection method. The study did not specify a detection methodology for reference. Mechanistic investigations further demonstrate that IL-17A promotes insulin resistance by potentially activating NF-κB and other signaling pathways, stimulating hepatic gluconeogenesis and adipose tissue lipolysis, and inducing serine phosphorylation of insulin receptor substrate-1 (IRS-1) in skeletal muscle [32]. Collectively, these findings position IL-17 as a crucial immunometabolic mediator linking psoriasis-related inflammation with the metabolic disturbances observed in diabetes (Figure 1). Consequently, targeting IL-17 may present novel therapeutic avenues for both psoriasis and diabetes.

## Chronic low-grade inflammation as a shared pathological basis

Chronic low-grade inflammation serves as a fundamental connection between psoriasis and T2DM. Persistent hyperglycemia promotes the accumulation of advanced glycation end-products (AGEs), which interact with their receptor RAGE on cell surfaces [4]. This interaction can activate NF-κB signaling, resulting in the increased expression of pro-inflammatory cytokines such as TNF-α, IL-6, and IL-1β [35]. The ensuing cascade amplifies oxidative stress and inflammation, impairs skin barrier function, and diminishes antimicrobial peptide production, thereby heightening the risk of infection. Notably, the psoriasis-associated protein psoriasin can also act as a RAGE ligand, further establishing the AGE-RAGE axis as a molecular link between psoriasis and diabetes [34].

Furthermore, pathway-level bioinformatic analyses reveal several shared signaling pathways in psoriasis and T2DM, including the NF-κB, necroptosis, NOD-like receptor, TNF, and Toll-like receptor pathways [36]. These interactions may exacerbate psoriatic inflammation in the context of diabetes [36]. Dysregulation of necroptosis and NF-κB not only contributes to β-cell injury in diabetes but may also intensify keratinocyte hyperproliferation and cutaneous inflammation in psoriasis [36].

Among cytokines, IL-1β plays a pivotal role in maintaining inflammatory circuits. It stimulates the production of downstream mediators such as IL-6 and TNF-α, thereby facilitating Th1 and Th17 immune responses [37]. In psoriasis, IL-1β directly contributes to epidermal inflammation and is closely associated with the IL-23/Th17 cell axis [37]. IL-6 is essential for various physiological functions, including glucose metabolism and glucagon-like peptide 1 (GLP-1) secretion [38]. However, its levels become pathologically elevated during chronic inflammation. Excess IL-6 promotes Th17 cell polarization, enhances keratinocyte proliferation, and disrupts skin barrier integrity [38]. Additionally, it impairs insulin sensitivity and β-cell function [38], positioning IL-6 as a crucial molecular link between the pathogenesis of psoriasis and T2DM.

IL-21, a significant cytokine produced by follicular helper T (Tfh) cells, peripheral helper T (Tph) cells, and some Th17 cells [39]. Both IL-21 and its receptor, IL-21R, are markedly elevated in psoriatic lesions and serum. The IL-21R is widely expressed on T cells, B cells, natural killer (NK) cells, and keratinocytes. Inhibition of IL-21 signaling suppresses keratinocyte proliferation and reduces the expression of interferon-γ (IFN-γ) and IL-17A [39]. These observations suggest that IL-21 enhances inflammation primarily by modulating T cell activity. Furthermore, IL-21 is critical for the development and maintenance of tissue-resident memory T (TRM) cells, which are significant contributors to psoriasis recurrence [39]. In diabetes, insulin resistance and elevated blood glucose levels promote the differentiation of Tfh cells and activate the mTOR-STAT3 pathway. Consequently, CD8+ T cells become more responsive to IL-21, and TRM cells persist in the skin [39]. This mechanism elucidates why T2DM may increase the risk of psoriasis or exacerbate its severity.

In addition to traditional cytokines, emerging biomarkers underscore the role of systemic inflammation in the intersection of psoriasis and T2DM. Glycoprotein acetylation (GlycA) serves as a composite marker of chronic inflammation and is frequently elevated in these patients, indicating persistent immune activation [30]. Intestinal fatty acid binding protein (I-FABP) acts as an indicator of gut barrier dysfunction and increased permeability, with elevated levels associated with insulin resistance and diabetes progression [30]. These findings emphasize the role of impaired barrier integrity and a diverse array of inflammatory mediators in linking psoriasis and T2DM.

## The cyclic GMP-AMP synthase (cGAS)-stimulator of interferon genes (sting) pathway: a mitochondrial stress-driven link

Animal studies indicate that diabetic mice, induced by a high-fat diet and streptozotocin, exhibit more severe IMQ-induced psoriasiform skin changes compared to non-diabetic controls [5, 40]. These diabetic mice present with elevated psoriasis area and severity index (PASI) scores, pronounced epidermal hyperplasia, and increased dermal immune cell infiltration. Furthermore, the lesions display heightened malondialdehyde levels and reduced expression of mitochondrial transcription factor A (TFAM) and OXPHOS complex proteins [33]. These findings suggest significant mitochondrial dysfunction and oxidative stress. It is essential to note that these models replicate psoriasiform dermatitis rather than true psoriasis, and that streptozotocin exerts direct toxic effects on β-cells. Subsequent experiments demonstrated that the selective STING inhibitor C-176 reversed these pathological changes in mice, with the treatment group showing decreased levels of NF-κB, TNF-α, IL-17A, and IL-23 in the skin [33]. The authors also analyzed skin samples from 22 patients, revealing that psoriasis patients with T2DM (*n* ═ 6) exhibited higher intensity of STING and phosphorylated interferon regulatory factor 3 (*P*-IRF3) staining compared to patients with psoriasis alone, those with T2DM alone, or healthy controls [33].

Clinical data further reveal that STING expression in psoriatic lesions from patients with T2DM is elevated relative to patients with T2DM alone and healthy controls [41]. This suggests a strong correlation between the STING pathway and disease severity. At the molecular level, co-stimulation of human keratinocytes with palmitic acid (PA) and IMQ induces mitochondrial membrane depolarization, resulting in the release of mitochondrial DNA (mtDNA) into the cytoplasm [33]. The cytosolic DNA is then detected by cGAS, leading to the production of the second messenger 2′,3′-cGAMP. Cell-based western blotting further demonstrates that this molecule activates STING, promoting its oligomerization and subsequent recruitment of TANK-binding kinase 1 (TBK1). TBK1 phosphorylates IRF3, resulting in increased expression of IFN-β and chemokines such as C-X-C motif chemokine ligand 10 (CXCL10). These chemokines facilitate the recruitment of Th1 cells and cytotoxic CD8^+^ T cells to the epidermis, amplifying local inflammation [33]. IFN-β can disrupt insulin receptor signaling, while CXCL10 may enhance the infiltration of autoreactive CXCR3^+^ CD8^+^ T cells into pancreatic islets, contributing to β-cell damage. Elevated glucose levels further intensify this signaling cascade [33, 41]. Concurrently, NF-κB activation promotes TNF-α and IL-23 expression, driving Th17 cell signaling. Collectively, these pathways establish a positive feedback loop that perpetuates psoriatic inflammation.

Moreover, commonly used metabolic drugs, including metformin and GLP-1 receptor agonists, may indirectly inhibit this pathway by enhancing mitochondrial function, reducing ROS generation, and limiting mtDNA release. These findings underscore the cGAS-STING axis as a critical inflammatory and metabolic hub, offering new avenues for integrated treatment of psoriasis in diabetic patients.

## Adipokine imbalance in obesity

Obesity is recognized as a state of chronic, low-grade systemic inflammation [42]. In high-fat diet-induced obesity models, epidermal γ δ T cells exhibit increased co-expression of CCR6 and IL-17A during the early phase of wound repair [24]. Conversely, in IMQ-induced psoriasis models, obesity does not elevate the overall IL-17 response, indicating that the polarization of epidermal γ δ T cells towards the IL-17-producing γ δ T17 phenotype is context-dependent. Local factors associated with obesity, such as elevated levels of CCL20 in the skin, persistent low-grade inflammation, and keratinocyte stress, may collectively lower the activation threshold of γ δ T cells [24].

Beyond immune cell modulation, obesity fundamentally alters the endocrine function of adipose tissue. Clinical studies reveal that psoriatic patients with metabolic syndrome (MS) exhibit significantly higher serum leptin levels and reduced adiponectin levels, shifting the adipokine profile toward a pro-inflammatory and insulin-resistant state [32]. Among these adipokines, resistin plays a particularly critical role; serum resistin levels in psoriatic patients are approximately twice those observed in individuals without metabolic comorbidities [43]. These levels correlate positively with PASI scores, fasting glucose levels, the homeostasis model assessment of insulin resistance (HOMA-IR) index, and inflammatory markers such as C-reactive protein (CRP), IL-6, and TNF-α[43]. Resistin acts synergistically with leptin and high mobility group box 1 (HMGB1) to create a positive feedback loop that perpetuates systemic inflammation and insulin resistance [43]. Mechanistically, resistin signals through the TLR4/CAP1 axis, activating multiple downstream pathways including NF-κB, JAK/STAT, and PI3K/AKT [43]. In the skin, this activation drives keratinocyte hyperproliferation, inhibits apoptosis, and promotes the expression of Th17 cell-associated cytokines such as IL-17, IL-23, and TNF-α, thereby exacerbating psoriatic inflammation. Circulating inflammatory mediators subsequently disrupt insulin signaling in peripheral tissues. Additionally, resistin enhances hepatic gluconeogenesis, stimulates lipolysis in adipose tissue, and induces serine phosphorylation of IRS-1 in skeletal muscle, collectively contributing to systemic insulin resistance [43]. Animal studies further support this mechanism; in IMQ-induced psoriasiform dermatitis combined with a high-sugar diet, mice display parallel elevations in blood glucose and serum resistin levels [43]. Anti-IL-17 therapy not only alleviates skin lesions but also restores glucose tolerance, suggesting that resistin may serve as both an early biomarker and a therapeutic target for psoriasis complicated by T2DM.

In addition to adipokines, lipid metabolic disturbances driven by caloric excess play a key pathogenic role. When subcutaneous adipose storage becomes saturated, excess free fatty acids (FFAs) accumulate ectopically in the liver, skeletal muscle, pancreatic β-cells, and even epidermal keratinocytes [44]. Elevated FFAs activate protein kinase C (PKC) and NF-κB signaling, inducing TNF-α and IL-6 production, thereby sustaining activation of the IL-23/Th17 cell inflammatory axis [44]. This dual effect exacerbates insulin signaling defects in metabolic tissues while perpetuating abnormal keratinocyte proliferation and pathological angiogenesis in the skin, establishing a vicious cycle between metabolic dysfunction and chronic cutaneous inflammation.

Elevated plasma triglycerides are associated with an increased risk of psoriasis, as demonstrated by both observational studies and Mendelian Randomization (MR) analyses. In the observational analysis, the multivariable-adjusted hazard ratio for psoriasis (ICD-10) per doubling of plasma triglycerides was 1.26 (95% confidence interval (CI) 1.15–1.39), with a corresponding causal odds ratio for incident psoriasis of 2.10 (95% CI 1.30–3.38) [45]. Hypertriglyceridemia may exacerbate psoriasis through the aforementioned mechanisms, and numerous studies highlight a robust association between psoriasis and components of metabolic syndrome, including obesity and dyslipidemia. These findings underscore the critical importance of lipid management. Future clinical practice should include routine screening for lipid abnormalities in psoriasis patients to facilitate early detection and timely intervention.

## Shared metabolic dysregulation

Studies utilizing the K14-VEGF-A transgenic mouse model of psoriasis have revealed that aging is correlated with a spontaneous exacerbation of skin inflammation, concomitant with systemic metabolic disturbances such as elevated fasting glucose, impaired glucose tolerance, and dyslipidemia [46]. This model provides compelling evidence that chronic skin inflammation can independently drive systemic metabolic dysfunction. Integrative gene expression analyses of psoriatic skin and diabetic pancreatic islets have identified three converging signaling pathways: phosphoinositide 3-kinase (PI3K)-protein kinase B (Akt), ras-related protein 1 (Rap1), and the cGMP-PKG pathway [15]. Further network analyses uncovered three core hub genes: small nuclear ribonucleoprotein polypeptide N (SNRPN), GNAS, and insulin-like growth factor 2 (IGF2) [15]. SNRPN is involved in systemic energy sensing, GNAS encodes the Gαs protein that mediates signaling in pancreatic β-cells and keratinocytes, and IGF2 promotes β-cell proliferation and epidermal hyperproliferation. These genes were consistently downregulated in independent validation cohorts and in peripheral blood samples from patients, indicating their potential as diagnostic markers for comorbidity and providing new molecular insights into disease co-occurrence.

In experimental psoriasis models with T2DM, phosphorylated AMP-activated protein kinase (*P*-AMPK) expression is significantly reduced, whereas keratinocyte growth factor (KGF) and signal transducer and activator of transcription 3 (STAT3) are upregulated [5]. As a crucial regulator of cellular energy metabolism, AMPK activation inhibits keratinocyte hyperproliferation and inflammatory responses [47, 48]. Both systemic and topical administration of metformin has been shown to reactivate AMPK signaling, downregulate KGF and STAT3 expression, reduce IL-17 receptor levels, and ultimately mitigate skin inflammation and hyperkeratosis [5]. These findings suggest that AMPK inhibition is a critical metabolic nexus linking psoriasis and T2DM, highlighting its potential as a therapeutic target.

Additionally, serum γ-glutamyl transferase (GGT) has emerged as an independent predictive marker for psoriasis, with stronger associations noted in patients with comorbid diabetes [49]. Under physiological conditions, GGT plays a role in maintaining intracellular antioxidant defenses by hydrolyzing extracellular glutathione (GSH). However, when GGT activity is abnormally elevated, it accelerates GSH depletion and promotes excessive ROS generation, leading to oxidative damage [50]. In IMQ-induced psoriasis models, increased ROS and diminished antioxidant enzyme activity are observed in skin lesions, with significantly reduced GSH levels in the serum and skin of psoriasis patients [51]. Clinical evidence supports the efficacy of antioxidant therapies in improving skin lesions [49]. This suggests that elevated GGT may contribute to skin inflammation and systemic insulin resistance through GSH depletion, ROS accumulation, and activation of key inflammatory pathways such as NF-κB and IL-23/Th-17 cells.

## Therapeutic management

The management of comorbid psoriasis and T2DM necessitates an integrated approach that addresses both skin lesions and systemic metabolic risk factors. Clinical observations indicate that inadequate glycemic control can diminish the effectiveness of psoriasis therapies. A retrospective study found that psoriasis patients with diabetes who received half-dose risankizumab exhibited lower PASI 75 response rates compared to their non-diabetic counterparts [52]. This emphasizes the necessity of maintaining good glycemic control to optimize dermatological treatment.

Both diseases appear to share a common inflammatory background, prompting clinical guidelines to increasingly advocate for multidisciplinary care. Collaboration between dermatology and endocrinology specialists is essential for managing both inflammation and metabolic dysfunction [53]. This integrated approach combines anti-inflammatory treatment with metabolic protection, as summarized in Table 1.

**Table 1:** Therapeutic strategies for the comorbidity of psoriasis and T2DM

**Agent**	**Specifics**	**Efficacy**	**Design**	**Notes**
**Biologics targeting the IL-23 or IL-17**				
Risankizumab	75 mg at weeks 0 and 4, then every 12 weeks for 52 weeks.	PASI 50/75/90/100 at week 40: 73.33/66.67 /63.33/46.67%; at week 52: 67.86/64.29/60.71/42.86%. Disease duration in the DM+ group was significant ly longer than in the DM– group (*P* = 0.0498; 0.0411)	Retrospective study; 30 patients [52]	The half-dose regimen (75 mg) still requires larger clinical studies to confirm efficacy.
Ixekizumab	Two injections (160 mg total) at week 0; 80 mg every 2 weeks to week 12; then 80 mg every 4 weeks to Week 60.	Similar PASI 75/90 response rates in patients with and without diabetes; delayed PASI 100 response in patients with diabetes.	RCT post hoc analysis; 564 patients [54]	No observed adverse effects on fasting glucose, HbA1c, or lipid profile.
Bimekizumab [55]	For 16 weeks.	Complete clearance of psoriatic lesions.	Case report	-
Guselkumab [56]	Two injections.	Complete resolution of all plaques, including those at injection sites.	Case report	Topical calcipotriol and betamethasone dipropionate are combined.
**Traditional hypoglycemic drugs with potential to improve psoriatic skin lesions**				
Liraglutide	-	Lower PASI (SMD --4.332, 95% CI --7.611 to --1.053, *P* ═ 0.01); lower fasting plasma glucose compared with baseline (SMD --0.341, 95% CI --0.679 to --0.004, *P* ═ 0.048).	Meta-analys is of 4 trials, 32 patients [11]	No significant change in HbA1c.
	Once daily before breakfast; titrated by 0.6 mg per week to a maximum of 1.8 mg; for 12 weeks.	Changes in PASI and DLQI from baseline were significantly greater in the treatment group (*P* < 0.05); HbA1c, HOMA-IR, and C-peptide decreased significantly from baseline (*P* < 0.05); Expression of IL-17, IL-23, and TNF-α in psoriatic skin was improved.	RCT, 25 patients [22]	Control treatment: Acitretin 30--50 mg/day, calcipotriol ointment, and conventional antidiabetic drugs
Semaglutide	For 12 weeks.	Median PASI decreased from 21 (IQR 19.8) at baseline to 10 (IQR 6) at week 12 (*P* ═ 0.002); IL-6 and CRP decreased (*P* < 0.05).	RCT, 31 patients [57]	-
	0.25 mg/week for 4 weeks, then 0.5 mg/week; maintenance dose 1 mg/week reached at week 16 and then continued.	PASI 8.0 (-76.0%); HbA1c 6.4% (from 7.9%); fasting glucose 124 mg/dL (from 162 mg/dL) at week 16. PASI 2.6 (-92.2%); HbA1c 5.4%; fasting glucose 98 mg/dL at month 10.	Case report [6]	Combined with metformin.
Metformin	Oral.	Psoriasiform dermatitis improved.	Animal study.	Some reports suggest that combined use with AGIs may increase psoriasis risk [58]
Dapagliflozin [59] (SGLT2i)	Topical.	Improved inflammator y markers and psoriatic lesions.	Animal study.	One study reported a higher risk of psoriasis in patients treated with SGLT2i compared with DPP4i (HR 1.08, 95% CI 1.03-1.13) [60]
Sitagliptin [61] (DPP-4i)	100 mg once daily (50 mg once daily in moderate kidney disease) for 24 weeks.	At week 24, the mean PASI change from baseline was --1.0 (95% CI --2.0 to 0.0), significantly greater in the sitagliptin + NB-UVB group than in the NB-UVB alone group (*P* ═ 0.044).	RCT, 118 patients.	With NB-UVB.
Pioglitazone [62](TZD)	15 or 30 mg once daily.	Marked reduction in PASI score in patients with psoriasis (WMD 2.68, 95% CI 1.41-3.94, *P* < 0.001).	Meta-analysis of 6 trials, 270 patients.	Some trials used combination therapy, not pioglitazone monotherapy.
**Other drugs**				
Apremilast [12] (PDE-4i)	For 52 weeks.	75.2% reduction in mean PASI score; ESR and CRP decreased. Improved blood glucose in patients receiving insulin and/or oral hypoglycemic agents.	Prospective observation al study, 137 patients.	Synergistic effect with insulin and/or hypoglycemic therapy.
Tacrolimus [63]	Initial dose 3 mg/day (0.05 mg/kg/day). For 2 weeks.	Skin lesions almost completely resolved.	Case report	Low dose, short course; no increase in blood glucose; improved kidney function observed.
Oral enzyme combination (OEC) [64]	2 tablets twice daily for 8 weeks.	Complete remission of skin lesions; slight reduction in HbA1c; decreased CRP.	Case report	Combined with exercise and dietary control.

Abbreviations: T2DM: Type 2 diabetes mellitus; PASI: Psoriasis area and severity index; DM: Diabetes mellitus; RCT: Randomized controlled trial; SMD: Standardized mean difference; CI: Confidence interval; DLQI: Dermatology life quality index; HOMA-IR: Homeostatic model assessment of insulin resistance; IQR: Interquartile range; AGIs: Alpha-glucosidase inhibitors; SGLT2i: Sodium-glucose cotransporter 2 inhibitors; DPP4i: Dipeptidyl peptidase-4 inhibitors; NB-UVB: Narrowband ultraviolet B; TZD: Thiazolidinedione.

### Biologics targeting IL-23 or IL-17

Biologics targeting IL-23, such as risankizumab, and IL-17, such as ixekizumab, not only clear psoriatic lesions and reduce PASI scores but also improve markers of subclinical atherosclerosis and vascular inflammation [52, 54, 65]. These agents confer both metabolic and immunological benefits and are often the first-line treatment for patients with T2DM requiring systemic therapy. However, diabetes may impede the speed of clinical remission. In a post-hoc analysis of a randomized controlled trial (RCT) involving ixekizumab administered over 60 weeks, PASI 75 and PASI 90 response rates were comparable between patients with normal blood glucose levels and those with diabetes. The analysis included 406 normoglycemic patients, 118 with prediabetes, and 40 with T2DM. Nonetheless, patients with diabetes demonstrated a significant delay in achieving complete clearance, with PASI 100 being reached at 60 weeks in the diabetes group compared to 12 weeks in the control group [54]. After adjusting for body mass index (BMI) and body weight, this delay remained significant, indicating that diabetes is an independent risk factor for slower lesion clearance. Importantly, long-term IL-17A inhibition does not exacerbate fasting glucose, HbA1c, or lipid profiles, suggesting a favorable metabolic safety profile. Case reports further illustrate that bimekizumab and guselkumab are effective in managing refractory psoriasis in patients with diabetes, including instances of the Koebner phenomenon at insulin injection sites [55, 56]. These observations reinforce the pivotal role of the IL-23/Th-17 cell axis in this comorbidity.

In addition to established biologics, novel immunomodulatory strategies are currently under development. One promising approach involves nano-based active immunization [37]. In a mouse model of psoriasis, supramolecular nanofibers containing IL-1β B-cell epitopes and a universal T-cell epitope (PADRE) can elicit strong and specific anti-IL-1β antibody responses. This vaccination reduces epidermal thickness and diminishes skin levels of IL-1β, IL-6, IL-17, and TNF-α, demonstrating efficacy comparable to direct IL-1β antibody therapy [37]. These findings suggest new avenues for precision immunotherapy in chronic inflammatory diseases.

### Glucose-lowering medications with immunomodulatory effects

Several glucose-lowering medications demonstrate potential benefits for patients with both psoriasis and T2DM [66, 67]. These agents not only improve glycemic control but also reduce immune inflammation, thus offering new avenues for combined therapies [68]. Among the most extensively studied are glucagon-like peptide-1 receptor agonists (GLP-1RAs). A meta-analysis involving 32 patients revealed that treatment with liraglutide resulted in significant reductions in the PASI score (SMD --4.332, 95% CI --7.611 to --1.053, *P* ═ 0.01) and fasting plasma glucose (SMD --0.341, 95% CI --0.679 to --0.004, *P* ═ 0.048) compared to baseline [11]. In another RCT involving 31 psoriatic patients with T2DM, a 12-week regimen of semaglutide combined with metformin achieved a PASI 90 response in nearly half of the participants (6 of 13) [57]. The median baseline PASI score in the semaglutide group was 21 (IQR 19.8), which decreased to a median of 10 (IQR 6; *P* ═ 0.002) after 12 weeks [57]. These patients also experienced reductions in serum IL-6 and CRP. Case reports indicate near-complete skin clearance with liraglutide or semaglutide, alongside improvements in the Dermatology Life Quality Index (DLQI) and glycated hemoglobin (HbA1c) [6, 55, 69]. However, current evidence is largely restricted to case reports and small RCTs, highlighting the need for larger trials to validate these findings. The immunomodulatory mechanisms of GLP-1RAs remain incompletely understood, but several potential pathways have been proposed.

First, weight loss associated with these medications may reduce the release of pro-inflammatory cytokines such as IL-6 and TNF-α from adipose tissue, thereby lowering systemic inflammation [57]. Second, immune cells express GLP-1 receptors; their activation can inhibit NF-κB signaling, reduce NLRP3 inflammasome formation, and downregulate mediators in the IL-23/Th17 pathway [6]. Third, GLP-1RAs may activate the AMP-activated protein kinase (AMPK) pathway, which alleviates oxidative stress and enhances insulin sensitivity, potentially creating an environment less conducive to psoriasis development [6, 70]. While most studies report beneficial outcomes, a few cases have documented new-onset or exacerbated psoriasis during GLP-1RA treatment [69]. This paradox underscores the complexity of GLP-1RAs’ immunomodulatory effects, which may vary based on individual genetic, immunological, or specific drug characteristics. Consequently, careful monitoring of skin symptoms during treatment is essential.

Metformin, a first-line therapy for T2DM, also exhibits benefits in psoriasis management [71]. Its mechanisms may involve AMPK pathway activation, which inhibits keratinocyte proliferation via the keratinocyte growth factor (KGF)/STAT3 axis and suppresses NLRP3 inflammasome-driven IL-1β release [5, 72]. Recent studies have linked its effects to modulation of gut microbiota. Metformin increases the abundance of Akkermansia muciniphila, with its protein Amuc-1100 alleviating psoriasiform dermatitis in mouse models by regulating indole-3-acetic acid, a microbial tryptophan metabolite [71]. This modulation leads to the suppression of the epidermal antimicrobial peptide S100A8. Notably, combining metformin with alpha-glucosidase inhibitors (AGIs) such as acarbose has been associated with an increased risk of psoriasis in a dose-dependent manner [58]. This risk may stem from alterations in gut microbiota composition, which can subsequently affect immune homeostasis.

Sodium-glucose cotransporter 2 (SGLT2) inhibitors are commonly prescribed for diabetes due to their cardiovascular protective effects and are currently being investigated for their influence on psoriasis. In IMQ-induced psoriasis mouse models, dapagliflozin ointments showed similar effects on erythema and scaling as clobetasol ointment at a concentration of 0.05%, without significant differences between groups. Dapagliflozin treatment also reduced inflammatory cytokines such as IL-17 and TNF-α, indicating potential inhibition of the Th17 pathway [59]. However, a large population-based, propensity score-matched cohort study comparing 550,195 T2DM patients treated with SGLT2 inhibitors to those receiving DPP-4 inhibitors found a higher incidence of new-onset inflammatory skin disease, including psoriasis, within five years of treatment initiation. The incidence in the SGLT2 inhibitor group was 3,767 out of 1,313,282 person-years, compared to 4,495 out of 1,732,071 in the DPP-4 inhibitor group (HR 1.08, 95% CI 1.03–1.13) [60]. These findings suggest that the immunomodulatory effects of SGLT2 inhibitors are multifaceted and may depend on the underlying disease state.

In a single-center RCT involving 118 patients with moderate psoriasis, the dipeptidyl peptidase-4 (DPP-4) inhibitor sitagliptin was found to enhance the anti-inflammatory effects of narrowband ultraviolet B (NB-UVB) phototherapy when used in conjunction [61]. At 24 weeks, the mean change in PASI from baseline was significantly greater in the sitagliptin plus NB-UVB group compared to the NB-UVB alone group (mean difference --1.0, 95% CI --2.0 to 0.0, *P* ═ 0.044). This improvement may result from the inhibition of excessive DPP-4 activity in the epidermis, which slows keratinocyte proliferation and modulates local T cell responses.

Thiazolidinediones (TZDs), such as pioglitazone, act by activating the peroxisome proliferator-activated receptor gamma (PPAR-γ) pathway [44]. This activation directly suppresses NF-κB signaling and reduces key cytokines, including TNF-α, IL-6, and IL-17. A meta-analysis of six RCTs (*n* ═ 270) demonstrated that pioglitazone was associated with a significant reduction in PASI scores among patients with psoriasis vulgaris (weighted mean difference 2.68, 95% CI 1.41–3.94, *P* <.001) [62]. In subgroup analyses, pioglitazone monotherapy proved effective for psoriasis vulgaris, while combination therapy with pioglitazone outperformed methotrexate, phototherapy, or acitretin alone [44, 62].

These findings illustrate that glucose-lowering medications exert diverse immunomodulatory effects on psoriasis through distinct molecular pathways. A careful evaluation of both benefits and risks is imperative for informing personalized treatment strategies that optimize outcomes for patients with T2DM and psoriasis.

### Other immunomodulatory drugs and strategies

Apremilast, an oral phosphodiesterase-4 (PDE4) inhibitor, enhances intracellular cAMP levels, effectively inhibiting pro-inflammatory cytokines such as IL-17, TNF-α, and IL-23, while promoting the anti-inflammatory cytokine IL-10 [12]. It is approved for the treatment of moderate-to-severe plaque psoriasis and psoriatic arthritis. A 52-week prospective observational study confirmed that apremilast significantly improved skin symptoms, with a 75.2% reduction in PASI scores, and decreased DLQI, nail psoriasis severity index (NAPSI), and joint-related scores [12]. Systemic inflammation markers, including CRP and erythrocyte sedimentation rate (ESR), also decreased significantly. Notably, the study observed a potential glucose-lowering effect, with diabetic patients receiving insulin or oral hypoglycemic agents experiencing a reduction in mean fasting blood glucose from 132 mg/dL to 121 mg/dL, although HbA1c showed no significant change. These results indicate that apremilast is effective in managing skin lesions and may provide additional metabolic benefits for psoriatic patients with metabolic abnormalities. Further investigation into its long-term efficacy and precise mechanisms is warranted.

Tacrolimus, a calcineurin inhibitor, primarily targets the NFAT signaling pathway, thereby inhibiting T-cell activation and the production of key inflammatory cytokines, including IL-2 and IFN-γ [73]. A case report highlighted its successful application in a complex comorbid patient with T2DM and stage 4 chronic kidney disease (CKD) who developed acute generalized pustular psoriasis (GPP) [63]. Following treatment with low-dose oral tacrolimus (3 mg/day), the patient’s skin lesions nearly resolved within two weeks, with remission maintained during dose reduction at three months and follow-up at six months. Diabetes control remained stable, showing no significant fluctuations in blood glucose levels. This case suggests that tacrolimus may represent an effective and manageable treatment option for complex comorbid patients with relatively stable metabolic conditions; however, further studies are needed to evaluate its immunologic mechanisms, long-term efficacy, and safety in this population.

Systemic inflammation can also be managed through non-pharmacological approaches. One case report documented significant reductions in CRP levels, resolution of arthralgia, and complete clearance of skin lesions in a psoriatic patient after eight weeks of treatment with an oral enzyme complex (OEC) containing bromelain, trypsin, and rutin [64]. The patient’s use of NSAIDs was reduced. Although symptoms recurred and CRP levels increased after discontinuation of the treatment, improvements were observed upon retreatment, indicating the sustained efficacy of this preparation in maintaining inflammatory remission. This presents a novel strategy for managing psoriasis and its associated comorbidities through modulation of systemic inflammation. Nevertheless, the long-term efficacy and specific immunomodulatory mechanisms of this approach in patients with diabetes and psoriasis warrant further validation in controlled studies.

## Conclusions and future directions

The comorbidity of psoriasis and T2DM is increasingly understood as a result of complex biological interactions rather than mere coincidence. Shared genetic susceptibilities, persistent low-grade inflammation, and immunometabolic crosstalk contribute to both disease processes. Central to this relationship are the IL-23/Th17 cell axis, NF-κB signaling, and mitochondria-derived stress responses, notably the cGAS-STING pathway, which link cutaneous inflammation with systemic insulin resistance and metabolic dysfunction. This mechanistic convergence positions psoriasis as a systemic disorder with significant metabolic implications, while concurrently implicating metabolic diseases like T2DM in exacerbating psoriatic inflammation.

This study has several limitations. First, psoriasis is a heterogeneous condition that encompasses various forms, including psoriasis vulgaris, pustular psoriasis, and psoriatic arthritis, each with distinct mechanisms and treatment responses. Second, population differences may constrain the generalizability of our findings. Third, publication bias is a possibility, particularly in small observational studies that report positive outcomes; while we aimed to include all available data, studies with null findings may be underrepresented. Furthermore, our narrative (non-systematic) approach means that some mechanistic and therapeutic conclusions remain provisional and context-dependent based on the type of evidence (preclinical, case-level, cohort, or randomized). These limitations should be considered when interpreting our results.

These insights advocate for a shift toward integrated, multidisciplinary management. Given the strong comorbidity between psoriasis and diabetes, along with the impact of diabetes on skin lesion recovery, clinicians should closely monitor diabetes-related symptoms in patients with psoriasis, facilitate timely screening, and ensure early diagnosis and intervention. Therapeutic strategies targeting shared molecular pathways, such as IL-17/IL-23 inhibitors and glucose-lowering agents with anti-inflammatory properties, including GLP-1 receptor agonists and metformin, offer the dual advantage of ameliorating both dermatological and metabolic symptoms. Additionally, comorbid conditions like CKD and CVD may influence treatment decisions in patients with psoriasis and T2DM. Certain systemic agents, including traditional glucose-lowering medications like metformin, require caution or dose adjustments in patients with impaired renal function. In patients with CVD, glucose-lowering agents that confer cardiovascular benefits, such as SGLT2 inhibitors, are preferred. Consequently, clinicians should account for the presence and severity of CKD and CVD in treatment selection and monitoring, coordinating care with nephrologists and cardiologists as necessary.

Future research should aim to elucidate the precise gene-environment interactions involved, identify robust biomarkers for patient stratification, such as resistin and STING, and develop novel therapeutics addressing both skin and metabolic pathologies concurrently. For instance, future studies could include GLP-1 receptor agonist randomized controlled trials in well-phenotyped psoriasis patients with predefined dermatological and metabolic endpoints, as well as mechanistic investigations of cGAS-STING in both pancreatic islets and skin. Ultimately, a comprehensive understanding of the interplay between psoriasis and T2DM is essential for advancing precision medicine strategies that enhance outcomes for individuals grappling with this challenging comorbidity.
